# Diagnostic Challenges in a Case of Refractory Severe Hypercalcemia Due to Splenic Sarcoidosis

**DOI:** 10.1210/jcemcr/luaf011

**Published:** 2025-02-04

**Authors:** Jeremy A Knott, Andrea R Horvath, Thaw D Htet

**Affiliations:** Department of Endocrinology, St George Hospital, Kogarah, NSW 2217, Australia; St George and Sutherland Clinical School, University of New South Wales, Sydney, NSW 2217, Australia; Department of Chemical Pathology, NSW Health Pathology, Prince of Wales Hospital, Sydney, NSW 2031, Australia; Department of Endocrinology, St George Hospital, Kogarah, NSW 2217, Australia

**Keywords:** refractory hypercalcemia, sarcoidosis, calcium disorders

## Abstract

Hypercalcemia is frequently encountered in clinical practice; however, sarcoidosis-induced hypercalcemia is relatively uncommon and requires careful evaluation, particularly when initial investigations are inconclusive or the hypercalcemia is refractory to standard treatment. We present a complex case of a 60-year-old female with chronic stage IV diabetic nephropathy who presented with acute severe asymptomatic hypercalcemia resulting from splenic sarcoidosis confirmed on splenic biopsy. Despite commencement of prednisone therapy, her hypercalcemia persisted. IV fluid therapy was complicated by fluid overload from chronic renal disease. Ketoconazole was trialed as second-line therapy with no initial improvement. Our case illustrates the diagnostic and therapeutic challenges associated with asymptomatic hypercalcemia attributed to systemic sarcoidosis on a background of chronic renal impairment. It underscores the importance of considering systemic sarcoidosis as a potential etiology in cases of acute PTH-independent hypercalcemia resistant to initial therapy.

## Introduction

Hypercalcemia is a common clinical abnormality, with 90% of cases attributed to either malignancy or primary hyperparathyroidism [1]. Other causes of hypercalcemia often require careful consideration, particularly when preliminary tests are inconclusive or calcium levels are refractory to initial therapy for the suspected underlying cause. Although hypercalcemia is a known metabolic complication of sarcoidosis, it is rarely a presenting manifestation and affects up to 10% of cases [2-5]. The primary mechanism of hypercalcemia is attributed to increased activity of vitamin D 1α-hydroxylase enzyme from activated granuloma macrophages, resulting in uncontrolled synthesis of 1,25-dihydroxyvitamin D3 (1,25(OH)_2_D) from 25-hydroxyvitamin D (25(OH)D) [6]. However, using vitamin D metabolites to assess disease activity remains challenging. We present a complex diagnostic and management case of sarcoidosis diagnosed on splenic biopsy in a 60-year-old White female who presented with asymptomatic severe hypercalcemia, refractory to conventional therapy, on a background of chronic stage IV diabetic nephropathy.

## Case Presentation

Our patient presented with acute severe asymptomatic hypercalcemia with paired corrected calcium of 3.50 mmol/L (14 mg/dL) (reference 2.1-2.6 mmol/L [8.4-10.4 mg/dL]) and PTH of 1.1 pmol/L (10.4 pg/mL) (reference 1.6-6.9 pmol/L [15.1-65.1 pg/mL]). She had normal 25(OH)D 68 nmol/L (27.24 ng/mL) (reference >49 nmol/L [19.6 ng/mL]) and mildly elevated 1,25(OH)_2_D 206 pmol/L (85.8 pg/mL) (reference 60-200 pmol/L [25-83.3 pg/mL]) on a background of chronically impaired renal function (creatinine 247 μmol/L [2.79 mg/dL], reference 45-90 μmol/L [0.5-1.0 mg/dL]). Other etiologies of acute PTH-independent hypercalcemia were investigated by measuring serum angiotensin-converting enzyme (ACE) activity as a nonspecific marker for granulomatous-inflammatory diseases, and PTH-related peptide (PTHrP) for ruling out hypercalcemia of malignancy. Both test results were undetectable. A 24-hour urinary calcium was low 2.0 mmol/24 hours (8 mg/24 hours) (reference 2.5-7.5 mmol/24 hours [10-30 mg/dL/24 hours]) with a normal urinary calcium to creatinine clearance ratio of 0.23 (>0.02).

## Diagnostic Assessment

**Figure 1.**
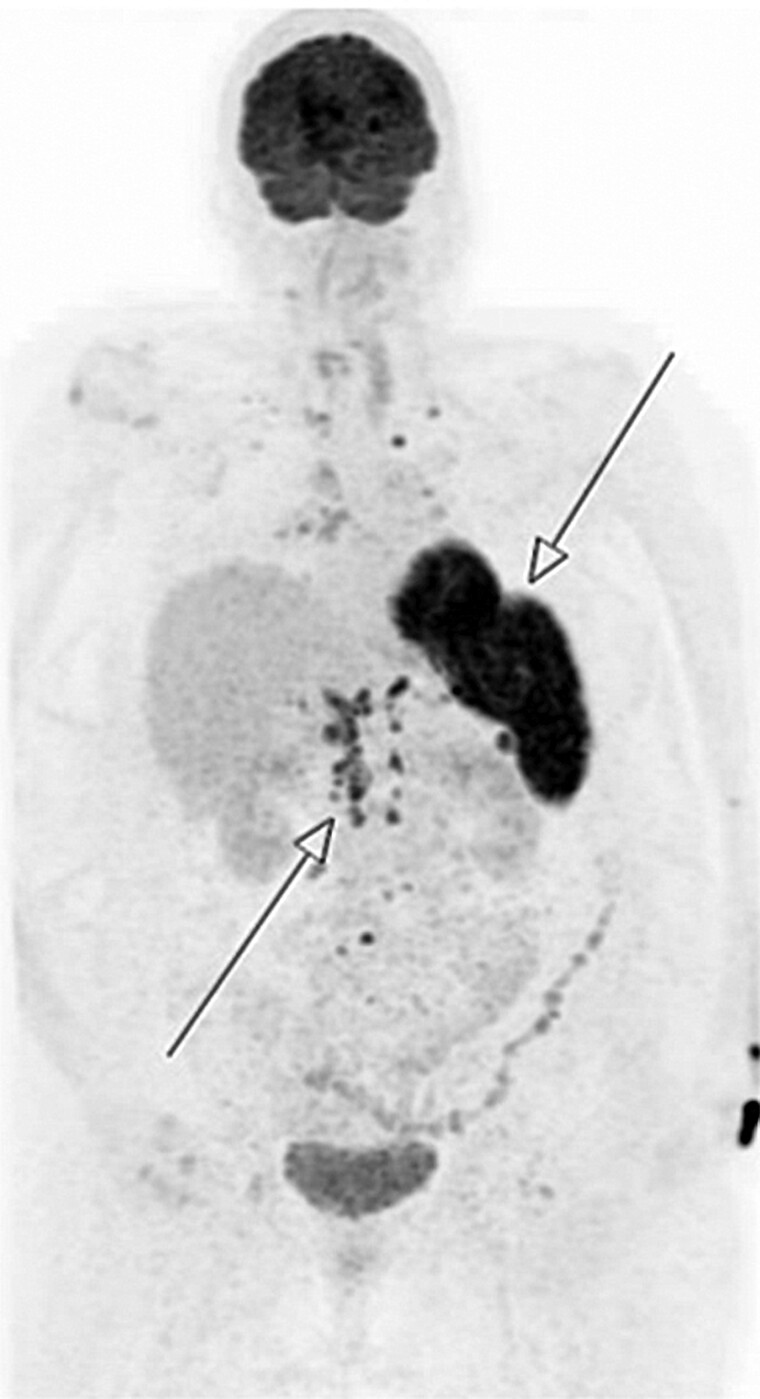
Positron emission tomography scan revealing fluorodeoxyglucose (FDG)-avid lymphadenopathy above and below the diaphragm coupled with FDG-avid uptake in the spleen.

While undergoing further investigations to ascertain the cause of her PTH-independent hypercalcemia, her corrected calcium remained persistently elevated above 3.0 mmol/L (12 mg/dL) for more than 5 weeks. This persisted despite treatment with IV fluids, IV 60 mg of pamidronate, multiple doses of subcutaneous denosumab at a reduced dose of 60 mg in accordance with our hospital's pharmacy formulary and clinician discretion, and 4 aliquots of 4 units per kilogram body weight of calcitonin. The patient subsequently underwent a positron emission tomography scan, which revealed fluorodeoxyglucose-avid lymphadenopathy and splenic uptake suggestive of lymphoma (Fig. 1). However, the subsequent splenic biopsy revealed sarcoid-like well-formed, diffuse granulomas and was subsequently diagnosed with extrapulmonary sarcoidosis.

## Treatment

**Figure 2.**
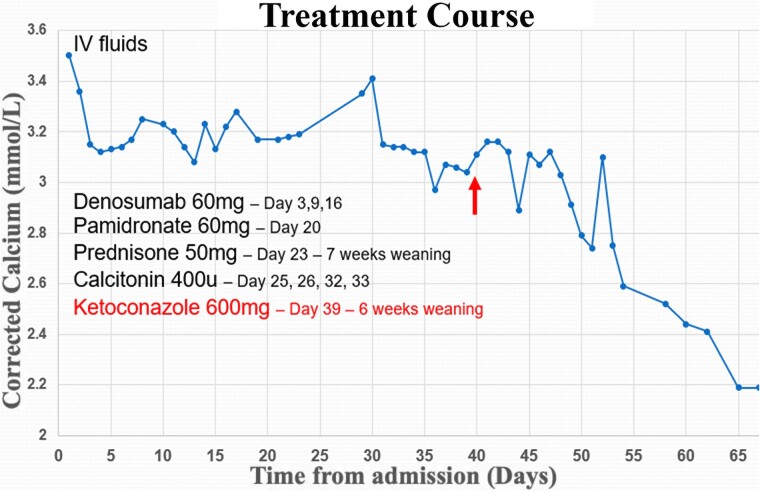
Corrected calcium levels (mmol/L, reference 2.1-2.6 mmol/L) over time (days from admission). The arrow indicates corresponding ketoconazole medical intervention.

Despite receiving prednisone 50 mg daily for 2 weeks, the severe hypercalcemia persisted and deteriorated to 3.41 mmol/L (13.64 mg/dL). Moreover, judicious use of required IV fluid therapy was limited because of her chronic renal impairment. Given that her hypercalcemia was not responsive to steroid therapy, coupled with a now normalized repeat 1,25(OH)_2_D of 161 pmol/L (67 pg/mL), the diagnosis of sarcoid-induced hypercalcemia was questioned. Conversely, although ACE activity was undetectable using the routine enzymatic assay while the patient was on an ACE inhibitor, ACE mass concentration using immunoassay showed high-normal values of 189 μg/L (11.3 U/L), (reference 37-221 μg/L [2.2-13.3 U/L]) despite steroid therapy. After multidisciplinary team discussion, second-line ketoconazole therapy was trialed. There was no initial improvement in serum calcium until 2 weeks of ketoconazole therapy (dosed at 200 mg daily before increasing to 600 mg daily), which improved her corrected calcium concentration to 2.74 mmol/L [10.96 mg/dL]) (Fig. 2).

## Outcome and Follow-up

After a total of 6 weeks of ketoconazole therapy and 7 weeks of prednisone her calcium normalized (2.20 mmol/L [8.8 mg/dL]). At the time of article submission, the patient has remained clinically and biochemically well. Her management plan is to continue ongoing calcium monitoring.

## Discussion

Hypercalcemia is a common clinical entity; however, sarcoidosis as an etiology for hypercalcemia is relatively uncommon. The incidence of hypercalcemia as the presenting symptom in sarcoidosis is 3% [7], which makes the diagnosis challenging particularly when hypercalcemia is refractory to conventional therapy. Our case is unique because there was no other clinical feature or radiographic evidence of systemic sarcoidosis, and the diagnosis was suggested only by splenic biopsy. Our case displayed important features: (1) markedly elevated serum calcium of 3.50 mmol/L in the absence of overt systemic sarcoidosis, (2) decreased 24-hour urinary excretion of calcium, (3) normal serum ACE activity and minimally elevated 1,25(OH)_2_D, and (4) hypercalcemia unresponsive to corticosteroid therapy.

Sarcoidosis is an idiopathic, multisystem, granulomatous disease, with extrathoracic splenic involvement occurring in up to 40% to 60% of cases [8]. Hypercalcemia in sarcoidosis is attributed to 3 potential mechanisms: (1) systemic extrarenal conversion of 25(OH)D to 1,25(OH)_2_D by 1α-hydroxylase produced by granulomatous macrophages and the consequential increase in intestinal calcium absorption; (2) granuloma production of PTHrP causing increased renal calcium absorption and bone resorption, similar to humoral hypercalcemia of malignancy; and (3) tissue-level conversion of 25(OH)D to 1,25(OH)_2_D by local granulomatous macrophages [9]. Interestingly, our case demonstrated only mildly elevated 1,25(OH)_2_D, in addition to undetectable PTHrP.

There have been similar case reports in the literature illustrating hypercalcemia with inappropriately normal 1,25(OH)_2_D in the setting of granuloma-forming disorders [10-12]. Proposed contributing factors for normal 1,25(OH)_2_D concentrations may include increased oral calcium intake, dehydration, and decreased calcium excretion, particularly in renal insufficiency [13], as seen in our patient with a baseline serum creatinine of 247 μmol/L (2.79 mg/dL). Our patient's low 24-hour urinary calcium excretion should be interpreted in the context of her impaired renal function [14] and reduced glomerular filtration [15]. A normal urinary calcium-to-creatinine clearance ratio of 0.23 (>0.02), along with the patient's clinical history, assisted in excluding familial hypocalciuric hypercalcemia.

The clinical utility of serum ACE activity in sarcoidosis is also limited because of poor sensitivity and specificity, with high ACE activity occurring in up to 75% of cases [16]. In addition, in our case, the patient was on an ACE inhibitor, which invalidated the ACE activity measurement. In such scenarios, measurement of the mass concentration of ACE is considered more useful. A comprehensive diagnostic approach in the evaluation of 1,25(OH)₂D-mediated hypercalcemia is necessary, particularly in atypical presentations with unclear etiologies [17]. Proceeding with a diagnostic biopsy in our case was imperative in confirming a diagnosis of sarcoidosis and in excluding potential alternative etiologies such as lymphoma or tuberculosis.

Corticosteroids are considered first-line therapy in treating sarcoidosis-associated hypercalcemia because of their effectiveness in treating granulomatous inflammation and rapidly correcting hypercalcemia from a relatively swift decrease in 1,25(OH)_2_D and calcium concentrations typically within 3 to 5 days [18]. As in our case of steroid-resistant sarcoidosis, ketoconazole may be effective in treating hypercalcemia because it inhibits 1α-hydroxylase required in 1,25(OH)_2_D synthesis [19]. Given ketoconazole is a potent inhibitor of steroidogenesis through its broad inhibition of cytochrome P450 enzymes, essential for cortisol synthesis in the adrenal cortex [20], it is therefore important to be mindful for adrenal insufficiency with prolonged use [21]. This is particularly relevant in our case as hypercalcemia may develop similar nonspecific symptoms to adrenal insufficiency, including gastrointestinal discomfort, reduced oral intake, and confusion [22]. Our patient's adrenal status was not assessed after completion of the 6 weeks therapy but failure to reassess cortisol status after prolonged therapy may lead to a delay in diagnosis of potential adrenal insufficiency. Additional steroid-sparing agents, such as infliximab, methotrexate, and azathioprine, may also help in treating refractory hypercalcemia by reducing granuloma formation [23].

Our case demonstrates an unusual presentation of extrathoracic sarcoidosis, presenting as prolonged, severe hypercalcemia with inappropriately normal or minimally elevated 1,25(OH)_2_D, which was refractory to conventional therapies including fluid therapy, antiresorptive therapies and steroid therapy. This case highlights the importance of multidisciplinary team input in managing uncommon causes such as sarcoidosis as a differential diagnosis for PTH-independent hypercalcemia, as it has a multisystemic and nonspecific presentation, with biopsy and radiographic findings as key to diagnosis. Timely recognition and appropriate treatment with second-line therapy may be necessary to avoid prolonged effects of first-line steroid therapy, particularly in cases with prolonged refractory hypercalcemia with multiple comorbidities such as type 2 diabetes and chronic renal failure.

## Learning Points

- Sarcoidosis-induced hypercalcemia is uncommon and presents diagnostic challenges.
- Persistent severe hypercalcemia can occur in sarcoidosis despite inappropriately normal or minimally elevated 1,25(OH)_2_D concentration.
- Medications such as ACE inhibitors and prednisone should be taken into consideration when interpreting ACE activity. Using ACE mass (immunoassay) rather than an enzymatic assay, may be considered more clinically reliable.
- Biopsy and radiographic findings are key to diagnosing sarcoidosis, even without overt systemic features.
- Refractory hypercalcemia requires timely recognition and consideration of second-line therapies, such as ketoconazole or other steroid-sparing agents. Multidisciplinary input is vital for managing complex cases effectively.

## Contributors

All authors made individual contributions to authorship. J.K., A.H., and T.H. were involved in the diagnosis and management of this patient. J.K. was involved in manuscript write-up and submission.

## Data Availability

Some or all datasets generated during and/or analyzed during the current study are not publicly available but are available from the corresponding author on reasonable request.

## Abbreviations

1,25(OH)_2_D: 1,25-dihydroxyvitamin D3
25(OH)D: 25-hydroxyvitamin D
ACE: angiotensin-converting enzyme
PTHrP: PTH-related peptide
